# Phospholipid/HP-β-CD Hybrid Nanosystems Amplify Neohesperidin Bioavailability via Dual Enhancement of Solubility and Stability

**DOI:** 10.3390/nano15110862

**Published:** 2025-06-03

**Authors:** Na Xia, Qian Zhou, Yanquan Liu, Dan Gao, Siming Zhu, Zuoshan Feng

**Affiliations:** 1 Key Laboratory of Biological Resources and Ecology of Pamirs Plateau of Xinjiang, College of Life and Geographic Sciences, Kashi University, Kashi 844000, China; conniexn@126.com (N.X.); zhouqian19900713@sina.com (Q.Z.); liuyanquan1823@163.com (Y.L.); 2 School of Food Science and Engineering, South China University of Technology, Guangzhou 510641, China; 3 Institute of Chinese Materia Medica, China Academy of Chinese Medical Sciences, Beijing 100050, China; dgao@icmm.ac.cn; 4 College of Food Science and Pharmacy, Xinjiang Agricultural University, Urumqi 830052, China

**Keywords:** neohesperidin, nano-liposomes, biopolymer conjugation, bioacceptability

## Abstract

Neohesperidin (NH), a bioactive flavanone glycoside, exhibits multifaceted pharmacological properties including antioxidant and anti-inflammatory activities. However, its clinical application is severely constrained by inherent physicochemical limitations such as poor aqueous solubility and instability under physiological conditions. To address these challenges, this study developed a dual-carrier nano-liposomal system through the synergistic integration of phospholipid complexation and hydroxypropyl-β-cyclodextrin (HP-β-CD) inclusion technologies. Two formulations—NH-PC (phospholipid complex) and NH-PC-CD (phospholipid/HP-β-CD hybrid)—were fabricated via ultrasonication-assisted ethanol precipitation. Comprehensive characterization using FTIR and PXRD confirmed the amorphous dispersion of NH within lipid bilayers, with complete elimination of crystalline diffraction peaks, indicative of molecular-level interactions between NH’s hydroxyl groups and phospholipid polar moieties. The engineered nanosystems demonstrated remarkable solubility enhancement, achieving 321.77 μg/mL (NH-PC) and 318.75 μg/mL (NH-PC-CD), representing 2.01- and 1.99-fold increases over free NH. Encapsulation efficiencies exceeded 95% for both formulations, with sustained release profiles revealing 60.81% (NH-PC) and 80.78% (NH-PC-CD) cumulative release over 72 h, governed predominantly by non-Fickian diffusion kinetics. In vitro gastrointestinal simulations highlighted superior bioaccessibility for NH-PC-CD (66.35%) compared to NH-PC (58.52%) and free NH (20.85%), attributed to enhanced stability against enzymatic degradation. Storage stability assessments further validated the robustness of HP-β-CD-modified liposomes, with NH-PC-CD maintaining consistent particle size (<3% variation) and encapsulation efficiency (>92%) over 30 days. Antioxidant evaluations demonstrated concentration-dependent DPPH radical scavenging, wherein nanoencapsulation significantly amplified NH’s activity compared to its free form. This study establishes a paradigm for dual-functional nanocarriers, offering a scalable strategy to optimize the delivery of hydrophobic nutraceuticals while addressing critical challenges in bioavailability and physiological stability.

## 1. Introduction

As a natural nutrient extracted from dried immature citron, neohesperidin (NH) has positive impacts on human health (e.g., anti-oxidation, anti-inflammation, anti-obesity) and can prevent several chronic diseases. However, neohesperidin is readily affected by oxygen, enzymes, high temperature, and gastrointestinal environment, resulting in limited applications in healthcare [1,2]. Recent research has demonstrated significant advancements in leveraging phospholipid-based strategies to enhance the oral bioavailability of both hydrophilic and lipophilic active substances. For example, mono- and di-glycerides/phospholipid (MDG/PL) complexes were shown to improve the relative bioavailability of lutein and DHA by 125.43% and 114.51%, respectively, in oil-based systems, highlighting their potential for nutrient delivery [3]. Furthermore, a soluble supramolecular complex of curcumin, developed by combining soybean phosphatidylcholine (PC) complexation technology with hydroxypropyl-β-cyclodextrin (HPβCD) inclusion technology, demonstrated a significantly enhanced dissolution rate and oral bioavailability [4]. These studies, along with recent reviews on phospholipid-mediated interfacial stabilization in drug delivery [5], collectively validate the versatility of phospholipids in addressing bioavailability challenges across diverse therapeutic applications. As an amphiphilic substance with high solubility in both organic and inorganic solvents, PC can serve as an effective bio-functional carrier to enhance drug solubility [6,7]. Additionally, good bio-compatibility allows PC to reach the cell cytoplasm without damaging lipid bilayers. Hence, the complexing of active substances with PC may facilitate delivery and absorption of active substances, thereby enhancing the bioaccessibility or pharmacological effects of PC [7,8]. PC extracted from soybeans has been the dominant PC for practical applications [5] owing to high content of phosphatidylcholine, which equips soybean PC with good compatibility and similarity with plasma membrane of mammals [1]. Besides its physiologically compatible pharmacokinetic and toxicological features, soybean PC also exhibits some clinically significant features, including hyperlipidemia resistance, liver protection, and heart protection [9,10]. However, PC complexes are typically used as pharmaceutical intermediates owing to low stability [7].

Liposome nanoparticles have become promising delivery systems for bioactive compounds due to their biodegradability, non-toxicity, environmental friendliness, and reproducibility [11]. Within the gastrointestinal environment, conventional liposomes can be unstable, especially upon exposure to enzymes and endogenous chemicals. Consequently, the absorption of orally administered materials is hindered, thereby limiting the practical application of conventional liposomes [12]. Since PC-β-cyclodextrin composites have successfully overcome the limitation of insoluble drugs [13], this study proposes a co-assembly strategy that integrates soybean phosphatidylcholine (PC) and hydroxypropyl-β-cyclodextrin (HP-β-CD) to encapsulate neohesperidin—a hydrophobic citrus flavonoid with low bioavailability. While previous studies have utilized phospholipids or HP-β-CD individually in hybrid carriers (e.g., transethosomes for transdermal delivery [14]), our approach leverages their synergistic interactions to enhance drug loading and stability. These PC/HP-β-CD complexes exhibit advantages such as cost-effectiveness, simple preparation, and promising market potential for nutraceutical applications. The supramolecular composites of neohesperidin and PC or HPβCD (NH-PC and NH-PC-CD) were characterized by TEM, dynamic light scattering, differential scanning calorimetry (DSC), and Fourier transform infrared spectroscopy (FT-IR) to systematically study the stability, antioxidant activity, and sustained-release properties of neohesperidin encapsulated in liposomes. The bioaccessibility of neohesperidin in liposomes was investigated by establishing an in vitro simulated digestion model.

## 2. Materials and Methods

### 2.1. Materials

HP-β-CD (purity ≥ 99.5%) was provided by Macklin Biochemical Co. Ltd. (Shanghai, China); neohesperidin (purity ≥ 98%) was purchased from Shandong Benyue Biological Technology Co., Ltd. (Dongying, Shandong, China); and soybean phosphatidylcholine (purity ≥ 99%) and cholesterol (purity ≥ 98%) were purchased from Aladdin Reagent Shanghai Co. Ltd. (Shanghai, China). All remaining reagents were of analytical (AR) grade.

### 2.2. Synthesis of PC-NH and NH-PC-CD Nanocomposites

Soy lecithin, cholesterol, and HP were used for synthesizing phospholipid and cyclodextrin NH liposomes by using the membrane dispersion method combined with ultrasonic technology [15]. First, PC, cholesterol, HP-β-CD, and NH (15:3:3:1 w/w) were mixed and dissolved in absolute ethanol. After complete dissolution, the solution was incubated in a water bath at 45 °C in a rotary evaporator to remove excess ethanol, followed by vacuum drying to obtain a lipid film.

Phosphate-buffered saline (PBS; 0.02 M, pH 7.4) was added, with the resulting solution maintained at 45 °C for half an hour to obtain neohesperidin, lecithin, and HP-β-liposome complex of CD (NH-PC-CD). For comparative studies, control formulations were prepared using identical methods with the following modifications: exclusion of HP-β-CD to generate neohesperidin-phospholipid complex liposomes(NH-PC), omission of neohesperidin to prepare HP-β-cyclodextrin-modified empty liposomes(PC-CD), and baseline empty liposomes lacking both neohesperidin and HP-β-CD(PC).

### 2.3. Average Particle Size and Polydispersity Coefficient

The diameter and zeta potential of nanocomplexes formulated in PC, PC-CD, NH-PC, and NH-PC-CD systems were quantitatively analyzed using dynamic light scattering (DLS) technology. Measurements were conducted at 25 °C employing a Zetasizer Nano ZS analytical system (Malvern Instruments Ltd., Malvern, UK) [16]. Prior to analysis, the samples were diluted to a relatively appropriate concentration.

### 2.4. Entrapment Efficiency of Neohesperidin

The entrapment efficiency of neohesperidin was determined at 283 nm by HPLC [17]. NH-PC and NH-PC-CD were subjected to a 10 min centrifugation at 4 °C and 10,000 rpm to separate the unpacked NH from the encapsulated one. The concentration of free NH in the resulting supernatant was then measured before calculating the encapsulation efficiency (EE) as follows:

$$\mathrm{EE}=\frac{\mathrm{Total}\;\mathrm{amount}\;\mathrm{of}\;\mathrm{NH}-\mathrm{free}\;\mathrm{amount}\;\mathrm{of}\;\mathrm{NH}}{\mathrm{Total}\;\mathrm{amount}\;\mathrm{of}\;\mathrm{NH}}$$

### 2.5. Characterization

#### 2.5.1. Nanocomplex Characterization

The nanocomplexes of PC, NH-PC, PC-CD, and NH-PC-CD were analyzed using a JEM-2100 transmission electron microscope (JEOL Ltd., Tokyo, Japan) at magnifications of ×150,000, following the sample preparation protocol described by Xiong et al. [18]. The measurement steps are as follows: fix the copper mesh with tweezers and use a small range (10 μL). Drop the pipette onto the copper mesh, and after 2 min, use filter paper to remove excess solution from the edge. Then, add 1% sodium phosphomolybdate solution for negative staining for 2 min. Then, use filter paper to remove excess solution from the edge and let it dry naturally. Determine its nanocomplexes using TEM.

#### 2.5.2. Fourier Transform Infrared (FTIR) Spectroscopy

Changes in the functional groups of NH and NH inclusion complex added with cosolvent were determined based on the infrared spectra of NH, PC, NH-PC, PC-CD, and NH-PC-CD, acquired using the KBr compression method. The method was the same as that of Xia et al. [1].

#### 2.5.3. Powder X-ray Diffraction (PXRD)

Changes in the characteristic peaks of NH, PC, NH-PC, PC-CD, and NH-PC-CD were determined as described by Xia et al. [1]. Briefly, a multi-position automatic injection X-ray diffractometer was used to acquire their PXRD patterns, with the major peaks and their corresponding intensities subsequently analyzed with Origin 9.1 software.

#### 2.5.4. Differential Scanning Calorimetry (DSC)

The thermal transitions of the samples (NH, PC, NH-PC, PC-CD, and NH-PC-CD) were analyzed using a DSC 214 Polyma instrument (NETZSCH, Selb, Germany). Approximately 5 mg of each sample was sealed in aluminum crucibles and subjected to a temperature program consisting of (1) initial equilibration at 25 °C for 5 min; (2) a first heating cycle from 25 °C to 300 °C at a rate of 10 °C/min; (3) cooling to 25 °C at 20 °C/min; and (4) a second heating cycle from 25 °C to 300 °C at 10 °C/min, with data from the second cycle being analyzed. All measurements were conducted under a nitrogen atmosphere with a constant flow rate of 50 mL/min.

### 2.6. Storage Stability

The storage stability of NH-PC and NH-PC-CD was assessed by placing the samples in a sealed container and storing them in darkness at 4 °C for 30 days, with samples taken every 10 days. The changes in particle size and EE% value during storage were studied.

### 2.7. Antioxidant Activity

Scavenging DPPH radical activity: The DPPH method [19] was used for radical scavenging activity with some modifications. Briefly, to 2.0 mL of 0.2 mM DPPH, 1.0 mL of varying concentrations (20, 40, 60, 80, or 100 μg/mL) of NH, NH-CD, or NH-PC-CD was added. Once fully dissolved, the samples were kept in the dark for 30 min at room temperature. After a 10 min centrifugation at 4000 rpm, the supernatant was collected to measure absorbance values at 517 nm with a spectrophotometer. For the control, solutions without samples were used. The experiments were conducted in duplicate, with DPPH radical scavenging activity calculated using the following equation:

$$\mathrm{scavenging}\;\mathrm{rate}\;(\%)=\frac{A_{\mathrm{control}}-A_{\mathrm{sample}}}{A_{\mathrm{control}}}\times 100$$

where Acontrol and Asample refer to the absorbance of the unspiked sample and the added sample, respectively.

Determination of ferric cyanide (Fe^3+^) reducing ability: The antioxidant ability of neohesperidin was assessed based on its reducing ability to Fe^3+^ in K_3_[Fe(CN)_6_] [19]. Briefly, a mixture of 1 mL of NH, NH-CD, and NH-PC-CD (20, 40, 60, 80, or 100 μg/mL), 2 mL of 0.2 mol/L PBS buffer (pH = 6.6), and 2 mL of potassium ferricyanide solution (1%) was heated for 20 min at 50 °C in a water bath. After cooling, the mixture was acidified with 2 mL of trichloroacetic acid solution (10%). After centrifugation, the supernatant (2 mL) was mixed with distilled water (2 mL) and then 0.1% FeCl3 solution (500 μL). The mixture was allowed to stand before recording absorbance values at 700 nm.

Reaction mechanism:

$$K_{3}\mathrm{Fe}\;(\mathrm{CN}{)}_{6}\;\frac{\mathrm{reductant}}{}\to \mathrm{Fe}\;(\mathrm{CN}{{)}_{6}}^{4-}$$

Fe(CN)_6_^4−^ + FeCl_3_ → K_4_[Fe(CN)_6_]_3_

Antioxidant activity was assessed using the FRAP (plasma iron reducing ability) and DPPH (2,2-diphenyl-1-picrylhydrazine) assays.

### 2.8. Research on In Vitro Release and Its Mechanism

The in vitro release study was performed on NH-PC and NH-PC-CD suspensions containing NH with dynamic dialysis [20]. A 5 mL suspension sample, sealed in a regenerated cellulose dialysis bag (MWCO 8–12 kD), was kept in a solution containing 0.5% (*w*/*v*) sodium lauryl sulfate and 20% (*w*/*v*) ethanol solution in PBS buffer (0.02 M, pH = 7.4) at (37 ± 0.5 °C) with gentle shaking (100 rpm) for the release experiment. At specific time points (0.5, 1, 2, 4, 6, 8, 10, 12, 24, 48, and 72 h), 0.5 mL of dialysate was taken and replaced with a similar volume of media. Each sample was analyzed using high performance liquid chromatography to calculate the accumulated release of NH. To investigate the release mechanism of neohesperidin liposomes, zero-order, first-order, Higuchi, and Peppas models were used to fit the release data.

### 2.9. Mucin Adhesion Study

The amount of mucin that could be adsorbed onto the surface of liposomes and the bioadhesive behavior of neohesperidin liposome formulations were assessed using porcine gastric mucin as the experimental model. Mix NH-PC and NH-PC-CD solutions with mucin solutions and rotate them at 37 °C for 1, 3, 6, 9, and 12 h, respectively. After adsorption, centrifuge the mixture at 10,000 rpm for 10 min, with the content of free mucin in the resulting supernatant assessed based on the periodic acid/Schiff staining method [21]. The amount of mucin adsorbed by NH-PC and NH-PC-CD liposomes is calculated from the difference between the mucin concentration in the solution after adsorption and the mucin concentration before adsorption. A calibration curve was also generated by repeating the above method using standard mucin concentrations of 0.1, 0.25, and 0.5 mg/mL. The content of mucin adhered per milligram of liposome is calculated based on the standard curve.

### 2.10. Simulate Gastrointestinal Digestion

To study how digestion affects the in vitro bioaccessibility of NH-P and NH-P-CD, a gastrointestinal tract (GIT) model was established [22]. All samples and solutions and samples were pre-incubated at 37 °C with shaking and kept at this temperature throughout the GIT process.

Gastric phase: Simulated gastric juice consisting of 3.2 mg/mL pepsin, 84 μM HCl, and 2 mg/mL NaCl were mixed with oral phase samples in a mass ratio of 1:1. After adjusting the pH to 2.5, gastric digestion was simulated by stirring the mixture for 2 h at 90 rpm and 37 °C [23].

Small intestine: A gastric phase sample was diluted with buffer (10 mM, pH 6.5) at a mass ratio of 1:1. The diluted digestive juice was kept at 37 °C for 10 min, and after adjusting its pH to 7.0, 50 mg/mL bile extract, 24 mg/mL trypsin (used to simulate intestinal fluid), and normal saline (7.5 M NaCl and 0.5 M CaCl_2_) were added. The pH, readjusted to 7.0, was maintained for 2 h to simulate the digestion period of the small intestine.

#### Measurement of Neohesperidin Bioaccessibility

**Figure 1:**
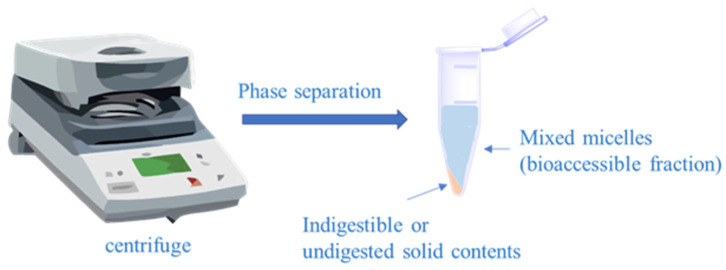
Schematic diagram of determination of bioacceptability by centrifugal separation.

Neohesperidin’s bioaccessibility was assessed after using the GIT model. Raw digestive juice was collected, and after a 30 min centrifugation at 15,000 rpm and 4 °C, the resulting supernatant was defined as the micelle fraction dissolved by neohesperidin, as shown in Figure 1. Neohesperidin was solubilized with methanol and determined by high performance liquid chromatography.

The transformation, bioaccessibility index, and bioaccessibility of neohesperidin can be calculated as follows:

$$\mathrm{Transformation}\;(\%)=100\times \frac{C_{\mathrm{Digesta}}}{C_{\mathrm{Initial}}}$$

$$\mathrm{Bioaccessibility}\;\mathrm{index}\;(\%)=100\times \frac{C_{\mathrm{Micelles}}}{C_{\mathrm{Digesta}}}$$

$$\mathrm{Bioaccessibility}\;(\%)=100\times \frac{C_{\mathrm{Micelles}}}{C_{\mathrm{Initial}}}$$

After passing through the simulated gastrointestinal tract, the neohesperidin bioaccessibility was calculated [24]. From each sample, 20 mL of digesta was taken and subjected to a 1 h centrifugation at 15,000 rpm, with the resulting supernatant assumed to be the micellar fraction soluble in NH. Then, NH concentrations in mixed micelles and digesta were determined, respectively. Based on that, the bioaccessibility of NH was calculated using the above formula.

Here, CMicelles and CDigesta refer to the concentrations of neohesperidin in mixed micelles and digesta at the end of GIT simulation, respectively, with CInitial representing the initial neohesperidin concentration.

### 2.11. Molecular Simulations

The three-dimensional (3D) structure of neohesperidin (NH) was retrieved from the PubChem database (PubChem CID: 442439) in SDF format. Structural optimization was performed using PyMOL 2.5. The PDBQT file of soy phosphatidylcholine (CP) was converted to PDB format for subsequent processing. AutoDockTools-1.5.7 was employed to prepare the molecular structures: (1) the PDB file of CP was imported, followed by removal of water molecules, addition of all hydrogen atoms, and computational processing; and (2) NH and CP were selected as the ligand and receptor, respectively. A docking box was defined to encompass the binding site region. Molecular docking was executed using AutoDock Vina with default parameters, and the resultant docking poses were generated. Post-docking analysis, including visualization of binding interactions, was conducted using the analysis module in AutoDockTools-1.5.7 and PyMOL 2.5 [17].

### 2.12. Statistical Analyses

All experiments were performed in triplicate. Data are presented as mean ± standard deviation (SD). Statistical analyses were performed using IBM SPSS Statistics 26, with one-way ANOVA for multi-group comparisons and unpaired Student’s *t*-tests for pairwise comparisons. Significant differences (*p* < 0.05) are denoted by different lowercase letters.

## 3. Results and Discussion

### 3.1. Characterization of Nano-Liposomes

**Figure 2:**
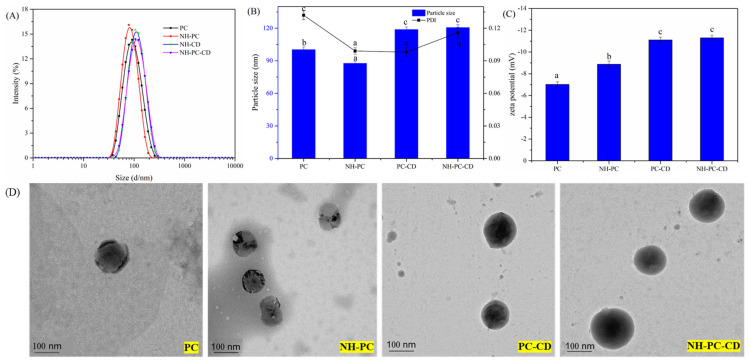
Particle size of PC, NH-PC, NH-CD, and NH-PC-CD (**A**) Z-Average particle size and polydispersity index (PDI) values of PC, NH-PC, NH-CD, and NH-PC-CD. Data expressed as mean ± SD (n = 3) a–c above the columns represent signiffcant differences with Particle size (*p* < 0.05); a–c above the columns represent signiffcant differences with PDI (*p* < 0.05) (**B**) Zeta potential of PC, NH-PC, PC-CD, and NH-PC-CD. Data expressed as mean ± SD (n = 3) a–c above the columns represent signiffcant differences with Zeta Potential (*p* < 0.05) (**C**) and Transmission electron microscopy (**D**).

The liposome systems prepared in this experiment include phospholipid nano-liposomes (PC), empty nano-liposomes (PC-CD) prepared by PC and HP-β-CD, nano-liposomes containing NH (NH-PC) and HP containing NH-β-CD conjugated nano-liposomes (NH-PC-CD), and the properties of all liposome samples are shown in Figure 2. Since the liposomes prepared by the film hydration method have problems of large particle size, unevenness, and poor stability [25], the liposomes prepared by the film hydration method were ultrasonically treated, and the particle size of liposomes was successfully reduced in this experiment. As observed, the particle sizes of PC, NH-PC, PC-CD, and NH-PC-CD were 79.2, 96.7, 102.9, and 132.2 nm, respectively, with PDI < 0.2 in all cases [26]. Ultrasonic treatment can significantly reduce the particle size of liposomes and maintain uniformity, which is due to the shear force generated when high-intensity ultrasonic waves flow through liposomes, resulting in the destruction of the multilayered vesicle structure and the formation of smaller vesicles [27]. The zeta potentials of PC, NH-PC, PC-CD, and NH-PC-CD were measured as −8.2 ± 0.31 mV, −9.5 ± 0.28 mV, −11.1 ± 0.27 mV, and −11.3 ± 0.25 mV, respectively (Figure 2C). The significantly higher negative zeta potential values of HP-β-CD-modified liposomes (PC-CD and NH-PC-CD) compared to their counterparts (PC and NH-PC) indicate enhanced electrostatic repulsion between particles, which suppresses aggregation and improves colloidal stability [27,28]. This aligns with the observed stability of NH-PC-CD during storage (Table 1), where minimal particle size variation and drug leakage were recorded. PC, NH-PC, PC-CD, and NH-PC-CD all have a uniform spherical structure with a particle size of 50–150 nm (Figure 2D), which aligned with the findings of dynamic light scattering. It is noteworthy that the high vacuum required for TEM observation may induce partial deformation of liposomes. However, the addition of HP-β-CD provided a stabilizing effect, as evidenced by the well-maintained spherical morphology of NH-PC-CD compared to NH-PC (Figure 2D).

Compared with PC and NH-PC, the carrier spherical structure of PC-CD and NH-PC-CD is more complete, and the morphology is better maintained. This is because the high vacuum required for TEM observation will lead to deformation of liposomes, and the sample added with HP-β-CD has a certain protective effect on liposomes.

**Figure 3:**
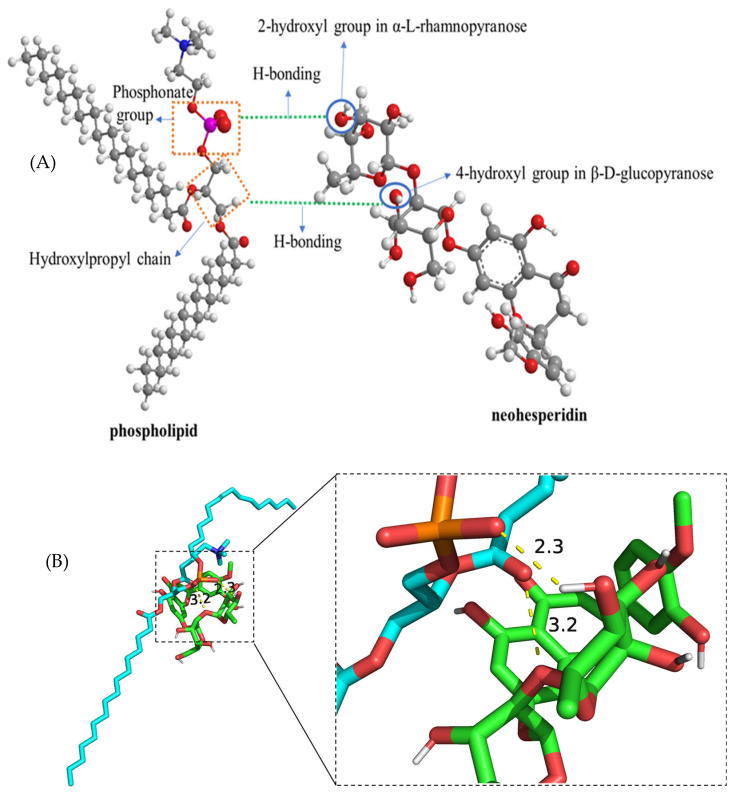
Hypothetical interaction between neohesperidin (NH) and nanolipo-somal phospholipid bilayer (**A**) and molecular docking visualization diagram (**B**).

Both PC and PC-HPβCD complexes can significantly improve the water solubility of NH. HPLC analysis revealed that the water solubility of neohesperidin in NH-PC and NH-PC-CD were 321.77 and 318.75 μg/mL, respectively. The loading rates of neohesperidin in NH-PC and NH-PC-CD were 64.35 ± 1.89 and 63.75 ± 2.04 μg/mg, respectively. The encapsulation rates of neohesperidin in NH-PC and NH-PC-CD were 96.52 ± 2.15% and 95.62 ± 1.17%, respectively. It was found that the two inclusion methods only slightly affected the encapsulation rates of neohesperidin liposomes. NH is a lipophilic compound composed of β-D-glucopyranose, α-L-rhamnopyranose, and hesperetin. The 2-hydroxyl ion in rhamnose can interact with the phosphate group of liposomes through hydrogen bonding(2.3 Å), while the 4-hydroxyl ion in the glucose group can form hydrogen bonding (3.2 Å)with the hydroxypropyl chain of phospholipids. Therefore, NH can be encapsulated in the lipophilic regions of liposomes [25,29]. For a clearer understanding, the hypothetical structure of the interaction between NH and nano-liposomes phospholipids is shown in Figure 3 [25].

### 3.2. FT-IR

**Figure 4:**
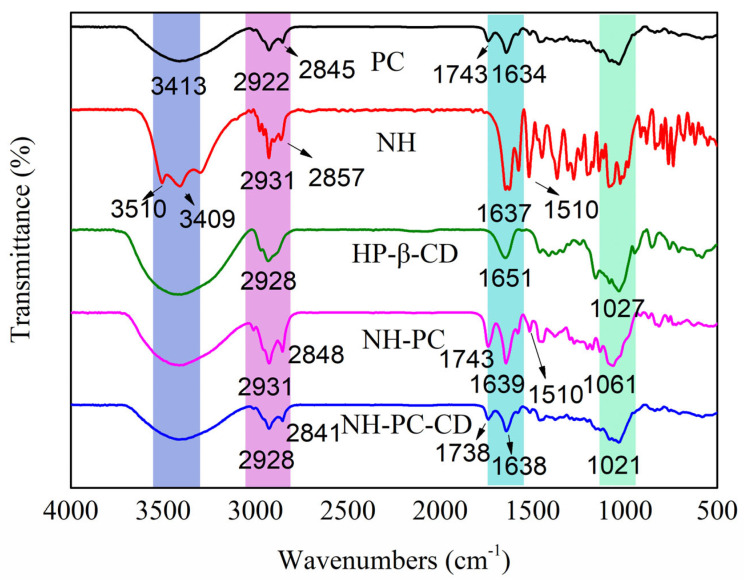
FTIR spectra: PC, NH, HP-β-CD, NH-PC, and NH-PC-CD.

The FT-IR spectra of PC, NH, HP-β-CD, NH-PC, and NH-PC-CD are provided in Figure 4. For NH, the peaks at 3510 and 3409 cm−1 correspond to -OH, while the signal at 2931 cm−1 could be attributed to the stretching vibration peak of alkane-C-H on B ring methoxy of neohesperidin. The stretching vibrations of C=O on the C ring carbonyl of neohesperidin and the C=C of benzene ring skeleton were indicated by peaks at 1637 cm^−1^ and 1510 cm^−1^, respectively. For PC, the peak at 1743 cm^−1^ is the stretching vibration peak of C=O, the peak at 1465 cm^−1^ is the bending vibration peak of -C-H, and the peaks at 1203 and 1080 cm^−1^ are stretching vibration peaks of P=O and P-O-C, respectively. For HP-β-CD, the characteristic peak at 3416 cm^−1^ corresponds to a wider characteristic band of -OH, the characteristic peak at 1651 cm^−1^ corresponds to the H-O-H deformation band of water molecules attached to HP-β-CD, and the peak at 1027 cm^−1^ is the stretching vibration peak of C-O-C. Compared with the characteristic spectra of NH and P, most of the characteristic peaks (e.g., 3510 cm^−1^, 3409 cm^−1^ and 1167 cm^−1^) of NH in the spectrum of NH-PC disappeared, while the characteristic peaks of P at 1743 cm^−1^ appeared, demonstrating the interaction of NH and PC in NH-PC. The spectrum of NH-PC-CD is basically similar to that of NH-PC. Compared with NH-P, the -C-H vibration peaks of NH-PC-CD shift from 2931 and 2848 cm^−1^ to 2841 and 3422 cm^−1^ respectively. The C = O stretching vibration peaks move from 1743 cm^−1^ to 1738 cm^−1^, and all the characteristic peaks of NH-P are weaker than those of NH--CD. This could be attributed to the fact that NH-P fitted into the cavity of HP-β-CD, thereby weakening the absorption intensity or causing its disappearance.

### 3.3. XRD

**Figure 5:**
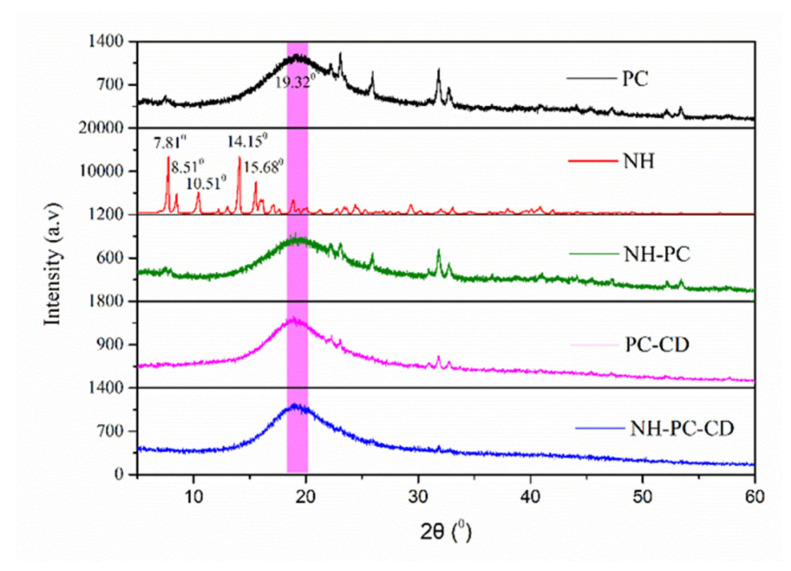
XRD spectra of PC, NH, NH-PC, NH-CD, and NH-PC-CD.

The physical state of neohesperidin in the delivery vehicle was characterized by XRD. Figure 5 shows XRD spectra of neohesperidin and neohesperidin complexes. The 2θ range of the typical peaks of NH is between 5° and 20°, and the peaks corresponding to the strong crystal diffraction peaks at 2θ are 7.81°, 8.51°, 10.51°, 14.15°, and 15.68°, which indicates that NH has a high crystal structure [24]. PC has a broad peak at 19.32°, without obvious crystal diffraction peaks, demonstrating that phospholipids are basically amorphous in structure. NH-PC mainly shows the wider diffraction peaks of PC, and NH-PC-CD shows that the diffraction peaks of PC are flatter than NH-PC, demonstrating that NH-PC is wrapped in HP-β-CD. Interestingly, the characteristic peaks of neohesperidin crystal form do not exist in the two composites, which could be attributed to interactions between neohesperidin and phospholipid molecules in the matrix to yield an amorphous composite [30]. Similar results also appeared in the relevant literature, wherein the encapsulation of neohesperidin in a polymer matrix transformed its crystalline structure into a non-crystalline one [17,24].

### 3.4. DSC

**Figure 6:**
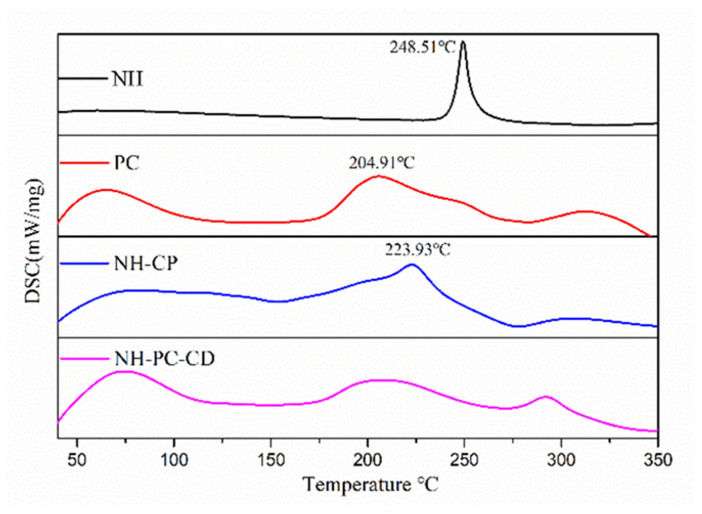
DSC thermograms of NH, PC, NH-PC complex, NH-PC-CD complex.

DSC melting enthalpy was used to confirm the formation of the complex. The characteristic peaks of neohesperidin were greatly reduced or disappeared in intensity and shifted to other peaks, demonstrating the successful formation of the composite. As shown in Figure 6, the thermal image of NH exhibited a sharp endothermic peak at 248.51 °C, which corresponded to the melting point of NH. Lecithin PC is amorphous, has no definite melting point in its differential scanning calorimetry, and has a large absorption peak at 204.91 °C. The DSC curves of NH-PC further showed that the characteristic peaks of NH at 248.51 °C are shielded, and the characteristic endothermic peak of lecithin also disappears, leaving only the characteristic curve of the composite itself, indicating complexity of NH and PC. The absence of NH’s melting endotherm in the first heating cycle—without requiring cooling data—conclusively demonstrates molecular-level dispersion within lipid carriers. Additionally, the DSC curves of NH-PC-CD and NH-PC are similar, demonstrating that NH-PC may be encapsulated within the cavity of HPβCD.

### 3.5. Storage Stability of Neohesperidin Liposomes

Owing to its intrinsic thermodynamic instability, liposome particles are readily exposed to agglomeration, degradation and melt, resulting in drug leakage during storage. Hence, storage stability is an important evaluation index to evaluate liposome stability. In this study, the particle size and EE% of NH-PC and NH-PC-CD liposomes during storage were investigated to determine their storage stability. NH-PC’s particle size increased from 87.66 nm to 102.99 nm during storage, while that of NH-PC-CD did not change much compared to NH-PC, only increasing from 120.71 nm to 122.92 nm, as shown in Table 1. After storage, the encapsulation efficiency of NH-PC and NH-PC-CD both tended to decrease, but the encapsulation stability of NH-PC-CD was better, and NH had only a small amount of leakage. Overall, NH-PC-CD has better storage stability compared with NH-PC at 4 °C. While the current formulation (NH-PC-CD) demonstrated improved storage stability compared to NH-PC (Table 1), further optimization strategies could be explored to enhance long-term stability for practical applications: functionalization with polyethylene glycol (PEG)-conjugated lipids could introduce steric stabilization effects, effectively suppressing interparticle aggregation through repulsive hydration layers. This approach has been validated in pH-responsive liposomal designs, where PEGylation significantly enhanced colloidal stability under physiological conditions [31].

### 3.6. In Vitro Antioxidant Activity of Nano-Liposomes

**Figure 7:**
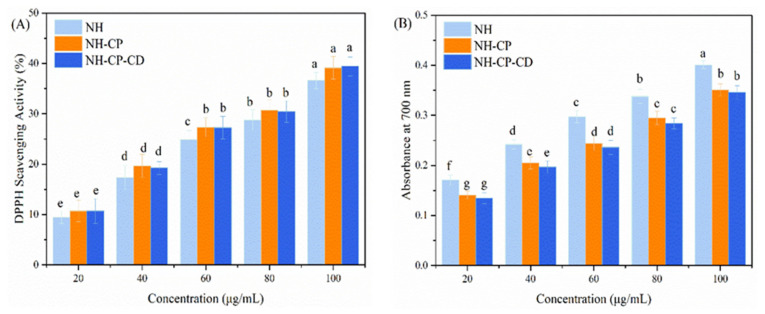
(**A**) DPPH free radical scavenging capacity and (**B**) Ferric reducing/antioxidant power (FRAP) of NH solution and nanoliposome in various concentrations. a–g above the columns represent signiffcant differences with DPPH and Absorbance at 700 nm (*p* < 0.05).

Currently, the in vitro antioxidant activity of neohesperidin encapsulated by nano-liposomes has not been studied yet. In the experiment, DPPH radical scavenging ability (organic solvent phase) and iron ion reducing ability (aqueous phase) were used as models to assess the in vitro antioxidant activity of neohesperidin liposome activity. Overall, the antioxidant activity of NH solution and NH liposomes (NH-PC and NH-PC-CD) had a significant relationship with the NH concentration (Figure 7A). As the NH concentration increased from 20 to 100 μg/mL, there was only a slight improvement in DPPH radical scavenging ability. However, the DPPH radical scavenging ability of NH increased after encapsulation by nano-liposomes at the same NH concentration. When the NH concentration was 20, 40, 60, 80, and 100 μg/mL, the scavenging capacity of NH solution was 9.47 ± 1.25%, 17.36 ± 2.38%, 24.91 ± 1.79%, 28.77 ± 2.01%, and 36.61 ± 1.62%, respectively, while the DPPH radical scavenging abilities of NH nano-liposomes (NH-PC) at the same concentration were 10.23 ± 2.15%, 19.66 ± 2.28%, 27.31% ± 1.89%, 30.72% ± 2.01%, and 39.14 ± 2.26%, respectively. The DPPH radical clearance ability of NH liposomes before and after HP-β-CD embedding was not significantly different. This indicated that inclusion complexation had no significant effect on the DPPH radical scavenging ability of NH. A similar situation was previously reported. Peres et al. [31] used gum arabic and maltodextrin to embed catechin and found that the scavenging ability of the DPPH radical was basically the same as that of free catechin solution, which indicated that the encapsulation of gum arabic and maltodextrin particles could maintain the antioxidant activity of catechin. In this experiment, NH liposomes showed higher antioxidant capacity compared with free NH, which was mainly related to the environment of NH. NH is a hydrophilic substance, and hence, it can be encapsulated in bilayers of liposomes, with significantly lower polarity than that of a water environment. However, DPPH has high hydrophobicity, so NH liposomes have higher DPPH radical scavenging ability. This aligned with the results of Niu et al. who found that curcumin liposomes had higher radical clearance than free curcumin [32].

The reduction ability of iron ions was determined based on absorbance values at 700 nm to reflect the reduction ability [33]. Figure 7B shows that the in vitro antioxidant activity of NH solution and NH liposomes (NH-PC and NH-PC-CD) is also dependent on the NH concentration. Under the same NH concentration, the iron ion reducing ability of NH-PC decreased compared with free NH, and there was no significant difference in the iron ion reducing ability of NH liposomes before and after HP-β-CD entrapment. Also, studies showed that the antioxidant activity of antioxidants would be affected to some extent after being embedded in a solid matrix of oil, chitosan, and glass powder. Donsì et al. [34] found that resveratrol and curcumin showed less ability to reduce iron ions after encapsulation in a liposome matrix than before. In this experiment, the iron ion reducing ability of NH decreased after being encapsulated by nano-liposomes and HP-β-CD. HP-β-CD also played a certain hindering role in the system, resulting in a lower iron reduction ability of NH liposomes.

### 3.7. In Vitro Release and Kinetic Studies

**Figure 8:**
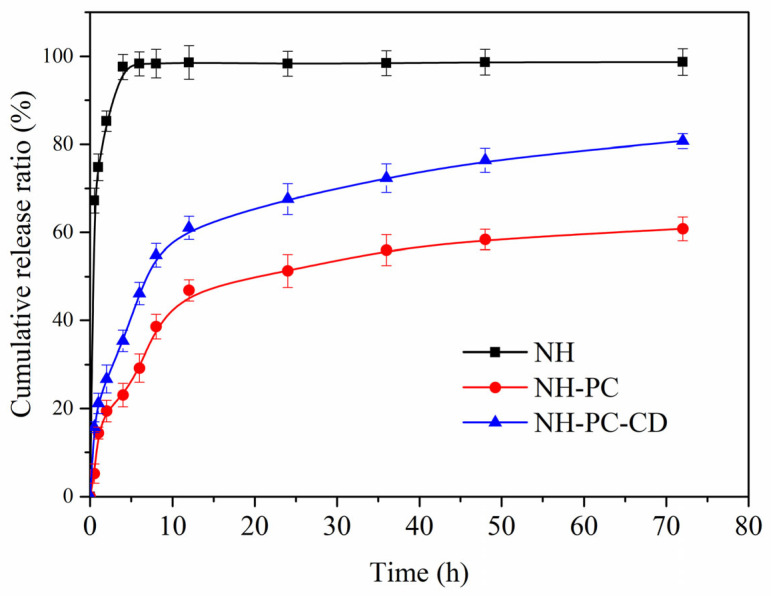
Drug release profiles of NH-PC and NH-PC-CD in PBS (pH 7.4).

The release behavior of neohesperidin was investigated using NH-PC and NH-PC-CD. Figure 8 shows the percentage of NH released from NH, NH-PC, and NH-PC-CD in PBS buffer (pH = 7.4). It was observed that the cumulative release rate of NH reached 97.61% within 4 h. Compared with NH, both composites decreased the release rate and release degree of NH-like in the release medium, which belonged to sustained release. Compared with NH-PC, NH-PC-CD showed a faster release rate in the release medium. Both composites showed fast reaction rates in the first 8 h, and the accumulated NH release exceeded 50% of the total release.

The release rates and degrees of both composite materials in the release medium decreased, which belonged to sustained release. Compared with NH-PC, NH-PC-CD has a faster release rate in the release medium. Both of the two composite materials showed a relatively fast reaction rate in the first 8 h, and the cumulative release of NH in both exceeded 50% of the total release.

In the first 8 h, 38.59 ± 2.81% of NH in NH-PC and 54.84 ± 2.66% of NH in NH-PC-CD were released. After 24 h, NH was released at a slower rate than that of the previous period, and the release continued until 72 h. At this time, the accumulated release rate of NH-PC reached 60.81 ± 2.69%, and the accumulated release rate of NH-PC-CD reached 80.78 ± 1.67%. In previous studies, it has also been shown that the drug with a lower release amount after sustained release for 72 h can be used as a potential drug for anti-tumor drugs. Wang et al. reported that the release rate of curcumin embedded in liposomes was lower than 50% in 24 h, and it had high bioaccessibility and cell activity [35]. Nguyen et al. studied the release rate of mPEG2000 cholesterol-modified pH-insensitive liposomes below 40% after 24 h and maintained this release rate, showing effective cellular uptake [28].

**Figure 9:**
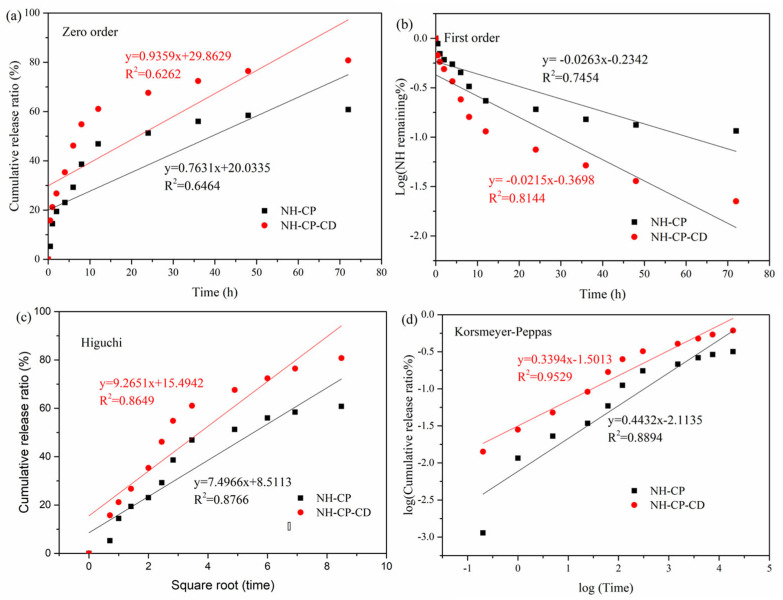
Release kinetics of NH-PC and NH-PC-CD fitted to four kinetic models: (**a**) zero-order kinetic model; (**b**) first-order kinetic model; (**c**) Higuchi model; and (**d**) Korsmeyer–Peppas model.

In order to further clarify the release characteristics of NH-PC and NH-PC-CD in vitro, the release curves in Figure 9 were respectively fitted to the following four models: zero-order, first-order, Higuchi, and Korsmeyer–Peppas. In this case, the model with an R^2^ value closest to 1 had the best correlation with the experimental results (best fit for the drug release curve) and was, therefore, considered as the optimal one. The results are shown in Table 2. According to previous reports, the most suitable drug release model could be determined from the top 60% of drug release [36]. A 72 h in vitro release study demonstrated that around 60% and 80% of the NH in NH-PC and NH-PC-CD were released, respectively. Therefore, the data of the two vector drug releases were fitted to the four models. Table 2 shows the kinetic release parameters and regression coefficients obtained for the four kinetic models.

(1) $$\frac{M_{t}}{M_{\infty }}=K_{0}t+C$$

(2) $$\ln\;(1-\frac{M_{t}}{M_{\infty }})=-K_{1}t+C$$

(3) $$\frac{M_{t}}{M_{\infty }}=K_{H}t^{0.5}+C$$

(4) $$\frac{M_{t}}{M_{\infty }}=K_{K}t^{n}$$

where Mt/M∞ refers to the amount released at moment t, K is the release constant, and n is the release index. The best fitting model was identified based on the highest R^2^ coefficient. According to Figure 9 and Table 2, the Korsmeyer–Peppas model was the best fit for the release results of NH in NH-PC and NH-PC-CD (R^2^ = 0.8894 and 0.9529, respectively), and the slope values are all less than 0.5 (n = 0.4432 and 0.3394, respectively). The kinetic model is often used to describe the Fick/non-Fick release of various complex phenomena [37]. The findings suggest that the NH release process of NH-PC and NH-PC-CD is similar to that of degraded polymer systems, and the release mechanism is diffusion-controlled.

### 3.8. Mucin Adhesion Study

**Figure 10:**
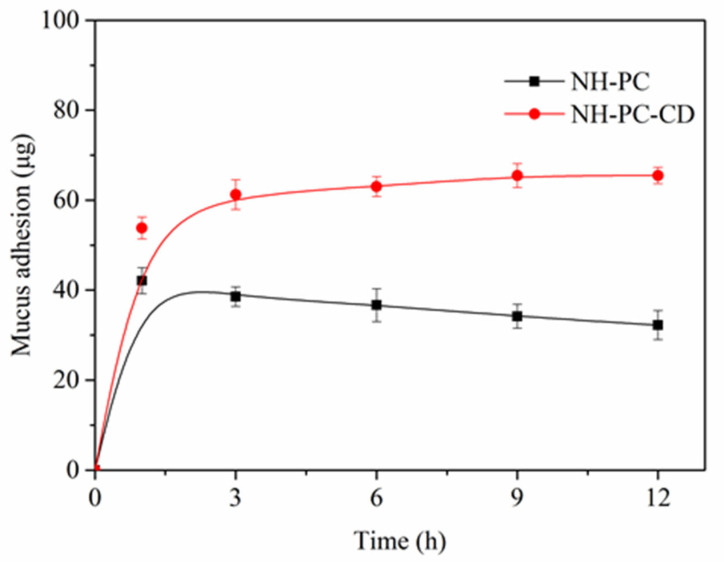
Adhesion kinetics of NH-PC liposomes and NH-PC-CD liposomes at a mucus concentration of 1 mg/mL.

Prolonging the residence time of oral nanomedicine delivery systems on the surface of gastrointestinal mucus holds significant importance. Through this approach the drug-loaded nanocarriers can sufficiently interact with intestinal epithelial cells, subsequently entering the lymphatic or circulatory system. Consequently, the adhesion of liposomes to intestinal mucus represents a critical step in extending their retention time at the absorption site, thereby enhancing the efficiency and effectiveness of oral drug absorption. By measuring the mucus absorption rate of NH-PC and NH-PC-CD liposomes at specific time points, the quantitative evaluation of the adhesion effect of NH-PC and NH-PC-CD on mucus was conducted. As shown in Figure 10, the mucin absorption rate of NH-PC and NH-PC-CD liposomes decreased gradually over time after 1 h. This phenomenon indicates that part of the NH-PC adsorbed on the mucus layer can be removed from the mucin adsorption site. In contrast, the mucin absorption rate of NH-PC-CD liposomes was higher within 12 h, indicating a more significant effect of prolonged residence time on the intestinal mucosa surface.

### 3.9. Bioaccessibility of Neohesperidin

**Figure 11:**
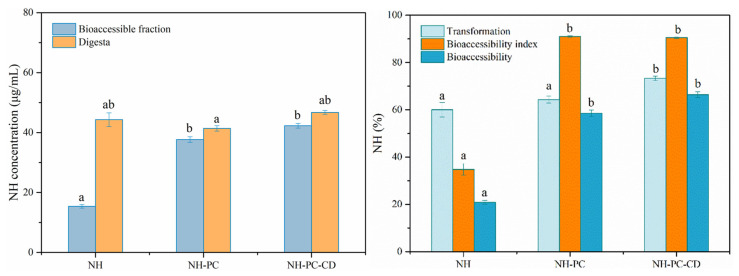
Neohesperidin bioaccessibility after in vitro digestion. a,b above the columns represent signiffcant differences with NH concentration and NH% (*p* < 0.05).

The transformation and bioaccessibility of neohesperidin were investigated by simulating gastrointestinal digestion in vitro. Specifically, NH, NH-PC, and NH-PC-CD were added to PBS buffer for in vitro simulated digestion. After digestion, neohesperidin in NH, NH-PC, and NH-PC-CD was chemically degraded (Figure 11), and the transformations were 60.01%, 64.32%, and 73.3%, respectively. According to Figure 10, PC-CD modification of NH can significantly improve the transformation of neohesperidin (*p* < 0.05), but a single PC modification did not significantly affect the transformation of neohesperidin. After PC-CD co-modification, the stability of the whole sample was increased, thus improving the transformation of neohesperidin in liposomes. Improving the stability of liposomes can reduce the contact between neohesperidin and gastrointestinal extreme environment and enzyme solution, thus inhibiting the degradation rate of neohesperidin.

The release rate of NH-PC and NH-PC-CD in the gastrointestinal tract digestion process is greater than 60%, and only the release rate is only 20% (Figure 10). This is because the hydrolysis of liposomes during gastrointestinal digestion will increase the release rate of neohesperidin in the emulsion, and the concentrations of micellized neohesperidin in NH-PC and NH-PC-CD are 37.65 and 42.29 μg/mL, respectively, with a high bioaccessibility index. However, due to the poor solubility of NH in digestive juice and the presence of particles, the concentration of micellized neohesperidin after digestion is only 15.38 μg/mL, with a low bioaccessibility index. Compared with the bioaccessibility of NH (20.85%), that of neohesperidin in NH-PC and NH-PC-CD were significantly higher at 58.52% and 66.35%, respectively (*p* < 0.05). Due to the low solubility of neohesperidin, it mainly exists in the form of undissolved crystals in gastrointestinal fluid, with low bioaccessibility. NH-PC and NH-PC-CD increase the solubility and stability of neohesperidin to improve its bioaccessibility. The results indicate that NH-PC and NH-PC-CD can significantly improve the bioaccessibility of neohesperidin.

## 4. Conclusions

The supramolecular complex PC-HPβCD with the advantages of both PC and HPβCD was prepared by combining phospholipid composite technology with HPβCD inclusion technology and demonstrated for the encapsulation of NH by the ultrasonic method and ethanol precipitation method. The average particle sizes of the as-prepared NH-PC and NH-PC-CD liposomes were small, the size distributions were uniform, and the morphology was spherical. Characterization by FT-IR and PXRD revealed that no diffraction peaks corresponding to NH were observed in the spectra of as-prepared composites, indicating that NH was completely dispersed in the liposome and NH molecules were exposed to intermolecular force with the phospholipid polar ends. Meanwhile, the solubility of NH-PC and NH-PC-CD increased by 2.01 and 1.99 times in comparison with that of neohesperidin, reaching 321.77 and 318.75 μg/mL, respectively. In vitro release tests showed that 60.81% and 80.78% of NH were released from NH-PC and NH-PC-CD, respectively, and non-Fick diffusion was the dominant release mechanism in both cases. In vitro simulation of gastrointestinal digestion demonstrated that NH-PC and NH-PC-CD significantly improved NH absorption, with NH-PC-CD also demonstrating greater bioaccessibility of NH liposomes than NH-PC, suggesting that the stability of NH liposomes was dependent on the composition of the carrier. In summary, nano-liposome carriers combined with PC and HP-βCD are promising as effective delivery systems to achieve the controlled release of lipophilic natural products.

## Figures and Tables

**Table 1:** Particle size and encapsulation efficiency of NH-PC and NH-PC-CD stored at 4 for 30 days. a–d above the columns represent signiffcant differences with Size and EE% (*p* < 0.05).

Liposomes	NH-PC		NH-PC-CD	
	Size	EE%	Size	EE%
Day 0	87.7 ± 1.3 a	96.5 ± 2.2 a	120.7 ± 1.7 a	95.6 ± 1.2 a
Day 10	93.0 ± 1.6 b	91.4 ± 1.9 b	121.1 ± 2.1 a	94.2 ± 1.0 a
Day 20	98.9 ± 2.1 c	87.7 ± 1.3 c	122.2 ± 1.9 a	93.8 ± 1.1 a
Day 30	103.0 ± 1.0 d	84.1 ± 2.1 d	122.9 ± 1.8 a	92.3 ± 1.2 a

**Table 2:** Rate constants and correlation coefficients of NH-PC and NH-PC-CD obtained through zero-order kinetic model, first-order kinetic model, Higuchi model, and Korsmeyer–Peppas model.

	Mathematical Models								
	Zero Order Model		First Order Model		Higuchi Model		Korsmeyer–Peppas Model		
	*K*_0_	*R*_0_^2^	*K*_1_	*R*_1_^2^	*K_H_*	*R_H_* ^2^	*K_K_*	*R_k_* ^2^	n
	37 °C								
NH-PC	0.763	0.646	0.0263	0.745	7.50	0.877	2.11	0.889	0.443
NH-PC-CD	0.936	0.626	0.0215	0.814	9.27	0.865	1.50	0.953	0.339

## Data Availability

The original contributions presented in the study are included in the article, and further inquiries can be directed to the corresponding author.

## References

[1] Xia N., Wan W., Zhu S., Liu Q. (2020). Preparation of crystalline nanocellulose/hydroxypropyl β cyclodextrin/carboxymethyl cellulose polyelectrolyte complexes and their controlled release of neohesperidin-copper (II) in vitro. Int. J. Biol. Macromol..

[2] Liu G., Yang J., Wang Y., Liu X., Guan L., Chen L. (2019). Protein-lipid composite nanoparticles for the oral delivery of vitamin B12: Impact of protein succinylation on nanoparticle physicochemical and biological properties. Food Hydrocoll..

[3] Huang S., Yin H., Tian F., Wang L., Wu W., Li X., Zhao Y., Wang X., Li Y., Terp M. (2025). Effects of mono-and di-glycerides/phospholipids (MDG/PL) on the bioavailability of lutein and DHA in rats: More effective for lutein. Food Biosci..

[4] Li Z.-L., Peng S., Chen X., Zhu Y., Zou L., Liu W., Liu C. (2018). Pluronics modified liposomes for curcumin encapsulation: Sustained release, stability and bioaccessibility. Food Res. Int..

[5] Drescher S., Blume A. (2025). The 8th International Symposium on Phospholipids in Pharmaceutical Research–An Update on Current Research in Phospholipids presented at the biennial Symposium of the Phospholipid Research Center Heidelberg. Eur. J. Pharm. Sci..

[6] Khan J., Alexander A., Ajazuddin, Saraf S., Saraf S. (2013). Recent advances and future prospects of phyto-phospholipid complexation technique for improving pharmacokinetic profile of plant actives. J. Control. Release.

[7] Pu Y., Zhang X., Zhang Q., Wang B., Chen Y., Zang C., Wang Y., Dong T., Zhang T. (2016). 20 (S)-Protopanaxadiol phospholipid complex: Process optimization, characterization, in vitro dissolution and molecular docking studies. Molecules.

[8] Ma J., Teng H., Wang J., Zhang Y., Ren T., Tang X., Cai C. (2014). A highly stable norcantharidin loaded lipid microspheres: Preparation, biodistribution and targeting evaluation. Int. J. Pharm..

[9] Prasad V., Gundermann S., Drozdzik M., Gunderman K. (2016). Essential phospholipids in fatty liver: A scientific update. Clin. Exp. Gastroenterol..

[10] Robert C., Couëdelo L., Vaysse C., Michalski M. (2020). Vegetable lecithins: A review of their compositional diversity, impact on lipid metabolism and potential in cardiometabolic disease prevention. Biochimie.

[11] Dai L., Li R., Wei Y., Sun C., Mao L., Gao Y. (2018). Fabrication of zein and rhamnolipid complex nanoparticles to enhance the stability and in vitro release of curcumin. Food Hydrocoll..

[12] Takeuchi H., Tahara K., Onodera R. (2016). Recent advances in particulate drug delivery systems: Oral, pulmonary, and ophthalmic administrations. Asian J. Pharm. Sci..

[13] Khatik R., Dwivedi P., Shukla A., Srivastava P., Rath S., Kumar Paliwal S., Dwivedi A. (2016). Development, characterization and toxicological evaluations of phospholipids complexes of curcumin for effective drug delivery in cancer chemotherapy. Drug Deliv..

[14] Singh J.P., Saini G., Tiwari G., Singh B. (2024). Nano-Formulation Approaches to Enhance Transdermal Drug Delivery-An Updated Review of Nanovesicular Carrier “Transethosomes”. Pharm. Nanotechnol..

[15] Tan Q., Liu S., Chen X., Wu M., Wang H., Yin H., He D., Xiong H., Zhang J. (2012). Design and evaluation of a novel evodiamine-phospholipid complex for improved oral bioavailability. AAPS PharmSciTech.

[16] Pujara N., Jambhrunkar S., Wong K., McGuckin M., Popat A. (2017). Enhanced colloidal stability, solubility and rapid dissolution of resveratrol by nanocomplexation with soy protein isolate. J. Colloid Interface Sci..

[17] Wang C., Xia N., Yu M., Zhu S. (2023). Physicochemical properties and mechanism of solubilised neohesperidin system based on inclusion complex of hydroxypropyl-β-cyclodextrin. Int. J. Food Sci. Technol..

[18] Xiong X.Y., Qin X., Li Z.L., Gong Y.C., Li Y.P. (2015). Synthesis, drug release and targeting behaviors of Novel Folated Pluronic F87/poly (lactic acid) block copolymer. Eur. Polym. J..

[19] Liu Y., Ying D., Cai Y., Le X. (2017). Improved antioxidant activity and physicochemical properties of curcumin by adding ovalbumin and its structural characterization. Food Hydrocoll..

[20] Wan K., Sun L., Hu X., Zijun Yan Z., Zhang Y., Xue Zhang X., Zhang J. (2016). Novel nanoemulsion based lipid nanosystems for favorable in vitro and in vivo characteristics of curcumin. Int. J. Pharm..

[21] Liu Y., Yang T., Wei S., Zhou C., Lan Y., Cao A., Yang J., Wang W. (2018). Mucus adhesion-and penetration-enhanced liposomes for paclitaxel oral delivery. Int. J. Pharm..

[22] Ribas-Agustí A., Martín-Belloso O., Soliva-Fortuny R., Elez-Martínez P. (2018). Food processing strategies to enhance phenolic compounds bioaccessibility and bioavailability in plant-based foods. Crit. Rev. Food Sci. Nutr..

[23] Zou L., Zheng B., Zhang R., Zhang Z., Liu W., Liu C., Xiao H., McClements D. (2016). Enhancing the bioaccessibility of hydrophobic bioactive agents using mixed colloidal dispersions: Curcumin-loaded zein nanoparticles plus digestible lipid nanoparticles. Food Res. Int..

[24] Xia N., Wan W., Zhu S., Wang H., Ally K. (2020). Synthesis and characterization of a novel soluble neohesperidin-copper (II) complex using Ion-exchange resin column. Polyhedron.

[25] Shishir M.R.I., Karim N., Gowd V., Xie J., Zheng X., Chen W. (2019). Pectin-chitosan conjugated nanoliposome as a promising delivery system for neohesperidin: Characterization, release behavior, cellular uptake, and antioxidant property. Food Hydrocoll..

[26] Jahn A., Vreeland W., DeVoe D., Locascio L., Gaitan M. (2007). Microfluidic directed formation of liposomes of controlled size. Langmuir.

[27] Isailović B.D., Kostić I.T., Zvonar A., Đorđević V.B., Gašperlin M., Nedović V.A., Bugarski B.M. (2013). Resveratrol loaded liposomes produced by different techniques. Innov. Food Sci. Emerg. Technol..

[28] Le N.T.T., Nguyen D.T.D., Nguyen N.H., Nguyen C.K., Nguyen D.H. (2021). Methoxy polyethylene glycol–cholesterol modified soy lecithin liposomes for poorly water-soluble anticancer drug delivery. J. Appl. Polym. Sci..

[29] Londoño-Londoño J., Lima V.R.D., Jaramillo C., Creczynski-Pasa T. (2010). Hesperidin and hesperetin membrane interaction: Understanding the role of 7-O-glycoside moiety in flavonoids. Arch. Biochem. Biophys..

[30] Leuner C., Dressman J. (2000). Improving drug solubility for oral delivery using solid dispersions. Eur. J. Pharm. Biopharm..

[31] Peres I., Rocha S., Gomes J., Morais S., Pereira M.C., Coelho M. (2011). Preservation of catechin antioxidant properties loaded in carbohydrate nanoparticles. Carbohydr. Polym..

[32] Niu Y., Ke D., Yang Q., Wang X., Chen Z., An X., Shen W. (2012). Temperature-dependent stability and DPPH scavenging activity of liposomal curcumin at pH 7.0. Food Chem..

[33] Benzie I.F., Strain J.J. (1996). The ferric reducing ability of plasma (FRAP) as a measure of “antioxidant power”: The FRAP assay. Anal. Biochem..

[34] Donsì F., Sessa M., Mediouni H., Mgaidi A., Ferrari G. (2011). Encapsulation of bioactive compounds in nanoemulsion-based delivery systems. Procedia Food Sci..

[35] Wang H., Luo J., Zhang Y., He D., Jiang R., Xie X., Yang Q., Li K., Xie J., Zhang J. (2020). Phospholipid/hydroxypropyl-β-cyclodextrin supramolecular complexes are promising candidates for efficient oral delivery of curcuminoids. Int. J. Pharm..

[36] Bettini R., Catellani P.L., Santi P., Massimo G., Peppas N.A., Colombo P. (2001). Translocation of drug particles in HPMC matrix gel layer: Effect of drug solubility and influence on release rate. J. Control. Release.

[37] Fathi M., Barar J., Erfan-Niya H., Omidi Y. (2020). Methotrexate-conjugated chitosan-grafted pH-and thermo-responsive magnetic nanoparticles for targeted therapy of ovarian cancer. Int. J. Biol. Macromol..

